# Race and Employment: The Historical Case of Head Coaches in College Basketball

**DOI:** 10.3389/fsoc.2020.00069

**Published:** 2020-10-08

**Authors:** Cornel Nesseler, Carlos Gomez-Gonzalez, Helmut Dietl, Julio del Corral

**Affiliations:** ^1^Business School, Norwegian University of Science and Technology, Trondheim, Norway; ^2^Department of Business Administration, University of Zurich, Zurich, Switzerland; ^3^Department of Economics and Finance, University of Castilla-La Mancha, Ciudad Real, Spain

**Keywords:** discrimination, executive labor market, race, college basketball, NCAA

## Abstract

This study analyzes how the number of Black coaches in college basketball has evolved since 1947. The analysis puts a focus on the time period after 1973 when regulatory requirements changed and a new Division was established. The change in the number of Divisions created distorted conditions and led to a significant difference in the number of Black coaches within Divisions. We trace a significantly lower number of Black coaches in Division 3 which is still visible 40 years later. The results are time consistent, not clustered geographically, and unrelated to specific institutions. Our results have policy implications for college sports as well as other industries with similar working conditions.

## 1. Introduction

In the US, Blacks^1^, as well as other minorities, face disturbing challenges in the labor market (see e.g., Jacquemet and Yannelis, 2012; Laouénan, 2017), which includes a limited number of Blacks in influential positions. This under-representation and the type of data available make empirical comparisons difficult in many industries^2^. Moreover, the pool of potential candidates is highly asymmetric due to the sizable share of Whites with a respective education (Arcidiacono and Koedel, 2014). Thus, an analysis of an industry with sufficient Blacks in leading positions (e.g., coaches in college basketball) is relevant to the literature.

The role of racial minorities in leading positions has evolved in recent decades, and is a challenging issue for societies. In this study, we use the term “racial minorities” as defined by Healey et al. (2018) and Cunningham (2019). Specifically, the term refers to a number of individuals who share a common characteristic and belong to a group that faces discrimination in society. In the US, all individuals who do not belong to the socially privileged group of White Americans represent racial minorities (e.g., African Americans, Asians, Hispanics, and Native Americans) and suffer from a relative disadvantage in relation to the other larger social group, especially in managerial positions.

Managers and leaders are relevant figures for the success of companies and organizations. By managing the available resources and coordinating a group of subordinates, they are responsible for the results. Previous research has identified similar roles for coaches in sports, team leaders, and managers, which include training and motivating team members, devising tactics, and managing objectives (Ladyshewsky, 2010). If discrimination affects leadership positions, the negative effect on productivity will be more noticeable as leaders influence several members within an organization. Because of the economic, social, and historical implications, this study focuses on the role of Black head coaches in college basketball.

Similar to executive managers in other industries, sports coaches in college programs hold a visible and influential position. Head coaches in National Collegiate Athletic Association (NCAA) college sports are important for the Universities and are usually among the highest-paid employees. In Division I Men's Basketball, coaches from big-time programs benefit from million-dollar contracts, far beyond the average salary of faculty members and University presidents (Benford, 2007). For example, at the University of California, Berkeley, in 2017 the three highest-paid employees were the football and basketball head coaches and the athletics director^3^. Broadcasting figures and revenues demonstrate the impact of college basketball in the US, where televised matches reach regional-, often national-wide audiences (Grimshaw et al., 2013). The NCAA signed a 14-year contract to sell the television broadcast rights for Division I Men's Basketball for $10.8 billion in 2010 (Brown et al., 2017).

In the US, basketball is an important part of the Black culture (Ogden and Hilt, 2003) and a large pool of sports professionals are involved and available to access influential positions. However, research reveals an imbalance. While the majority of players are Blacks—64.3% between 2007 and 2010 (Harper et al., 2013)—, the number of Black coaches is comparably lower. If college basketball had a fair degree of racial equity—same number of Black athletes and coaches—, we would expect a share of Black coaches close to 48%. However, this imbalance is not only present in college basketball. The Institute for Diversity and Ethics in Sports–TIDES–(www.tidesport.org) reveals that Black coaches are also underrepresented in college football (2.7–5.7% since 1995, 4.9% in 2017) and college baseball (0.4–1.2% since 1995, 0.5% in 2017)^4^. It is important for research to examine why a sport with a significant cultural value for the Black community provides the majority of players but not the majority of coaches, even when experience as a player is a key determinant of coaching performance in basketball (Goodall et al., 2011).

The organizational structure of companies and institutions influences the possibilities of minorities to enter the executive labor market. Arrow (1998) and Darity and Mason (1998) discuss that race has historically served as a gatekeeping mechanism that controls access (and promotion) to managerial positions. Specifically, in NCAA college sports, Hawkins (2013) notices that athletic institutions are predominantly managed by Whites, whose preferences might prevent other minorities from reaching leadership positions. Hawkins describes how these institutions exploit athletic abilities while neglecting much needed academic abilities, which are necessary for coaches. Even though most institutions support and applaud their Black athletes, the same institutions refrain from hiring Black coaches (Brooks et al., 1996).

Regan (2014) supports that claim, stating that internal personnel (e.g., athletic directors) who are overwhelmingly White, have an important stance in the hiring process and act as gatekeepers. This might be amplified by hiring personnel who “make the connection concerning Black coaches and individuals comprising lower rankings in societies” (Agyemang and DeLorme, 2010, p. 44). Thus, institutional gatekeeping mechanisms might be equally applicable in college basketball. Singer et al. (2010) examine the hiring process for college football. They find that race matters when candidates are assessed. Performing a mock hiring process, Regan (2014) confirms that White coaches are more likely to be hired, all else being equal.

Black coaches also face barriers to developing a career after being hired. Cunningham et al. (2001) describes how normative pressures (e.g., the lack of role models) are an important reason Black coaches tend to leave their job earlier than White coaches. Additionally, the lack of social capital in the respective occupation (e.g., social networks) has a significant negative influence on Black coaches' careers (Day and McDonald, 2010). Cunningham (2020) provides a multilevel model to explain the under-representation of racial minorities in coaching positions in the US, which includes the case of the NCAA. The model establishes micro-level factors such as personal identity and self-limitation behaviors (e.g., Cunningham and Singer, 2010; Steward and Cunningham, 2015); macro-level factors such as institutional racism, preferences, and political climate (e.g., Stewart and Garcia-Prieto, 2008; Hylton, 2012); and meso-level factors such as biased decisions and organizational culture and policies (e.g., Borland and Bruening, 2010; DuBois, 2015).

Organizational policies are especially important for the representation of racial minorities in leading positions and are overlooked in the literature. Cunningham (2020) shows that there is a lack of empirical research on this matter. DuBois (2015) is one of the few researchers who focuses on the impact of policies. By empirically analyzing the effect of the Rooney Rule on racial diversity among National Football League (NFL) coaches, she finds a positive effect. This rule is a diversity-related policy, which is expected to improve the role of racial minorities. However, other historical policies that are not diversity-related may still have an impact on the role of minorities by shaping the structure of an organization and changing the incentives for the members of the majority group. The results from our study shed light on this issue and challenge further research to build upon them.

This study uses data covering the period 1947–2015 to examine the evolution of Black coaches in college basketball. The analysis distinguishes between all college basketball NCAA Divisions, which have different missions, regulations, and requirements for participants, and includes teams' geographical locations.

First, the organization model, based on three different Divisions with uneven performance standards, provides an opportunity to examine racial issues at different competitive levels. Divisions 1 and 3 are on opposite ends regarding financial status, athletic scholarships, number of athletes, sports facilities, and impact. For example, Division 1 membership is reserved for institutions with sufficient resources to afford high-level competition that reaches large audiences, whereas Division 3 institutions are not allowed to offer any athletic scholarships for competition. Division 2 stands somewhere between the two organizational models (Grant et al., 2014). The Division System subsection provides further details on these differences.

Second, the geographical information allows us to test historical racial differences in employment by race between southern and northern states. To further examine the characteristics of colleges and Universities (including financial data) that explain the number of Blacks, we restrict our data to the period 1987–2015 and estimate regression models that include the Blinder-Oaxaca decomposition. The results allow us to add empirical evidence to the historical discussion on differences between southern and northern states regarding racial discrimination (Wilson, 1996; Kuklinski et al., 1997; Wright and Esses, 2017).

Finally, the study examines the influence that the introduction of Division 3 in 1973 had on the employment of Black coaches. Although establishing causal relationships after policy changes is complicated (Shertzer et al., 2016), it is important to analyze the impacts. In professional football, DuBois (2015) analyzes the impact of a soft affirmative policy (the Rooney Rule) on the role of racial minorities in executive leadership, and in NCAA institutions, Carroll and Humphreys (2000) examine the (un)intended effect of Title IX on athletic departments. To the best of our knowledge, no research has analyzed the historical share of Black coaches in college basketball after the modification of the Division system in 1973. The analysis of this change has organizational and research implications as non-diversity-related policies may also affect the representation of racial minorities in influential positions.

The rest of the manuscript is organized as follows. First, we provide historical background information on college basketball. Second, we outline the data available in our analysis. Third, we empirically examine the evolution of Black coaches since 1947 and the influence of the structural change that occurred in 1973. Finally, we discuss the results, implications, and limitations of the study, and provide our conclusions.

## 2. Historical Background

### 2.1. The Beginnings of College Basketball

College teams first participated in annual championships against non-colleges (1915) and later in intercollegiate championships (1922). The set-up of the intercollegiate tournaments changed frequently (1950, 1953, 1975, 1980, 1985, 2001, and 2011) and since 1939 has been organized by the NCAA (Crowley, 2006).

Although basketball gained popularity among Black students, most major colleges were reluctant to include Black players on their teams^5^. After 1947, the share of Black athletes at predominantly White colleges steadily increased from 1% in 1948 to 34% in 1975 (Yetman et al., 1982). In the following decades, legal, social, and political changes improved the situation for Black athletes and students at colleges (Davis, 1994). Nonetheless, several authors have shown that Black college basketball players still face racial stereotypes (Davis, 1994; Lapchick, 1995; Love and Hughey, 2015) and are exploited by athletic departments (Leonard, 1986; Van Rheenen, 2013).

Basketball coaches have a prominent role in representing their colleges and are under constant observation (Becker and Wrisberg, 2008). Black coaches have been active in Division 2 and Division 3 since these divisions started in 1947 and 1973, respectively. Historically, however Division 1 was the slowest Division to introduce the first Black coach, Will Robinson (Illinois State University), in 1970. The first Black coach in our data, before the introduction of the Division system, is Byrd D. Crudup, who coached North Carolina Central in 1927.

**Figure 1:**
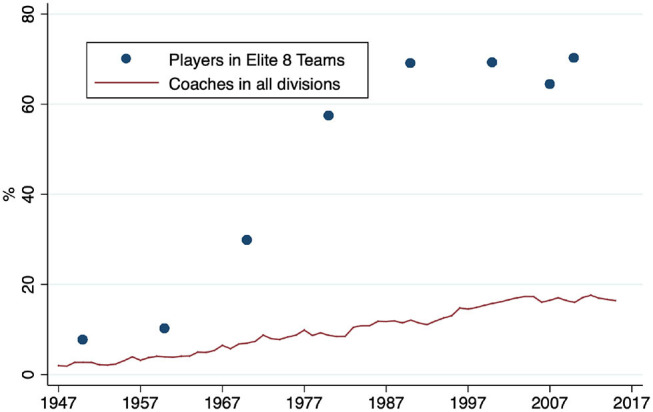
Share of Black players and coaches.

In NCAA Men's Basketball, no rule prevents Black coaches from being hired. However, the number of Black coaches is very limited when compared to Black players. Figure 1 shows the evolution of Black players (blue dots) and coaches (red line). While the number of Black players sharply increased during the analyzed period, the number of coaches improved only moderately. Currently, more than 60% of players in the Elite-8 teams are Blacks, while the share of Black coaches is below 20%^6^. This imbalance is surprising, as expert knowledge from a top playing career increases the success of basketball coaches in the US (Goodall et al., 2011).

### 2.2. The Division System

The Division system in NCAA Men's basketball started in 1947^7^. Institutions could choose to enter Division 1 or 2, or to not enter the NCAA Division system. Since 1973, colleges have also been able to join Division 3. Previous members of Division 2 either stayed in their division or joined one of the other divisions. Before 1973 the main difference between Division 2 and Division 1 was that schools were categorized based on their size. One reason the NCAA added another division was because of “the increasing difficulty of maintaining a level playing field between smaller-budget schools and those with major athletics programs” (Crowley, 2006). Another reason was the pressure from other college sports organizations (namely the National Association of Intercollegiate Athletics) resulted in the creation of another division that might be attractive for schools from other organizations (Katz and Seifried, 2014).

Table 1 shows the number of institutions that switched Divisions from 1972 to 1973^8^. The results demonstrate that the new Division 3 received most of its members from former Division 2 institutions. A smaller number come from institutions who previously participated in Division 1 or outside the Division system. We include a list of all institutions that switched from Division 2 to Division 3 in Appendix (Table 7).

**Table 1:** Division split up 1973.

**Year**		**Division 1**	**Division 2**	**Division 3**	**Outside D. system**
1972		139	268	/	180
1973		150	158	133	165
Net change 1973	Division 1	/	17	/	3
	Division 2	6	/	/	9
	Division 3	2	101	/	22
	Outside D. System	0	6	/	/

**Figure 2:**
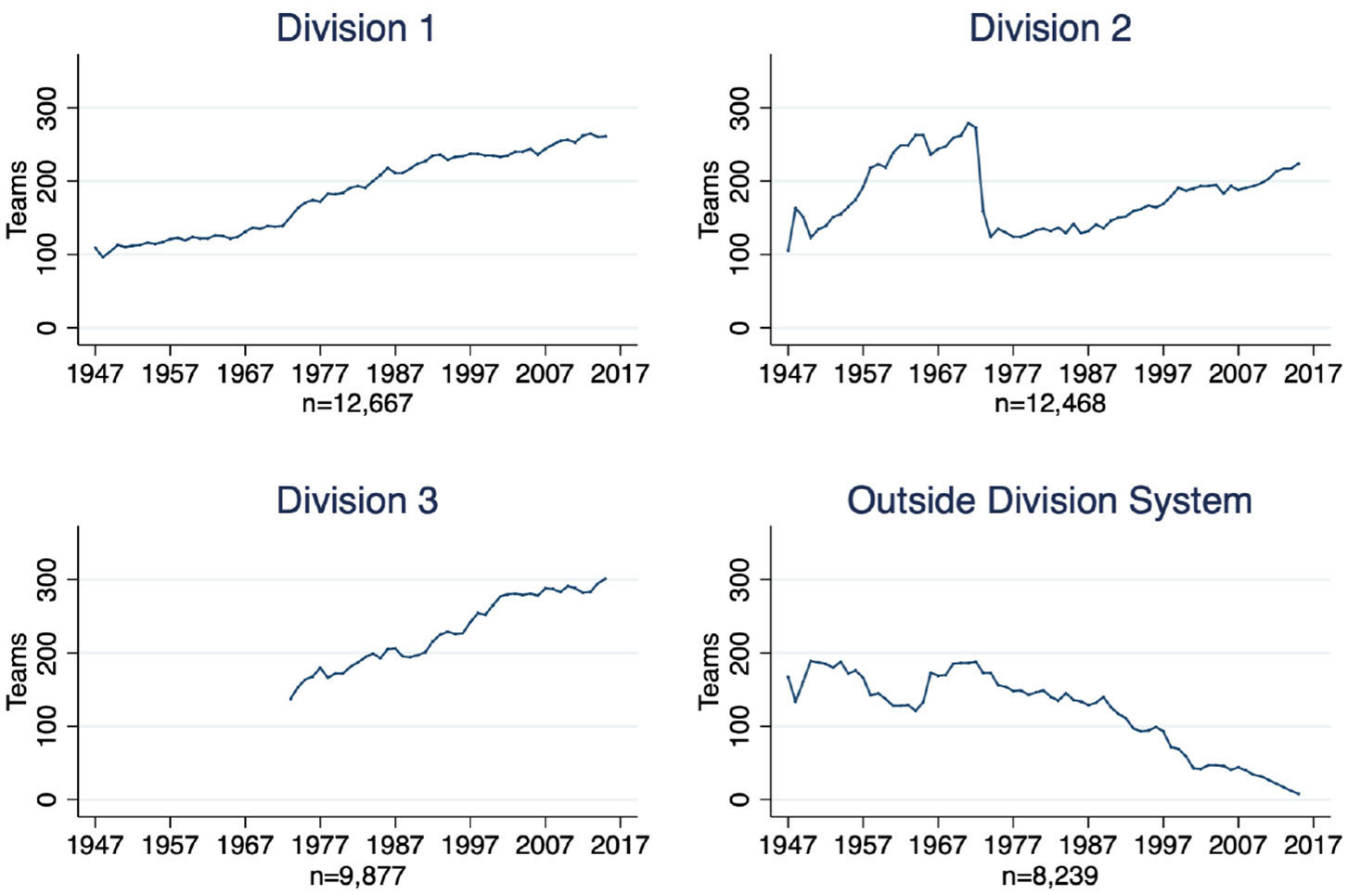
Total number of teams by Division (per season).

The creation of Division 3 influenced the composition of the divisions but not the total number of teams. Several teams in Division 2 switched to another division or outside the Division system. Since then, the total number of teams competing in the NCAA has steadily increased. Figure 2 shows how the number of teams evolved in every division and outside the Division system^9^. The largest reduction of teams was in Division 2 after the 1973 creation of Division 3.

The distinction between the divisions in NCAA basketball is because of the different governance conditions among the colleges and Universities. In the current Division system, Division 1 and Division 3 are asymmetric regarding financial status, athletic scholarships, number of athletes, and sports facilities. Grant et al. (2014) specify differences in the Division system. While Division 1 is reserved for institutions with enough resources to afford high-level competition, Division 3 institutions are not allowed to offer any athletic scholarships. Division 2 teams are a mix, where athletes receive partial financial scholarships and local or in-state quotas may apply^10^. The number of undergraduate students who are enrolled in sport activities is considerably larger in Division 1 institutions compared to Division 2 and 3 institutions (Grant et al., 2014). The number of participating students also influences market size and media attention (Woods, 2015)^11^.

The social impact of the athletic programs also differ in the divisions, for example, educational development of the student athletes. Woods (2015) argues that Division 3 institutions that compete locally are more likely to focus on raising academic standards and reducing expenses, while Division 1 institutions need to maintain a highly competitive performance to attract media attention and economic resources. Finally, Division 1 and 2 institutions must fill out an annual self-study guide. The aim of the guide is to help institutions comply with rules, regulations, and finances. It also includes a section about cultural diversity.

The duties and remits of coaches also differ in the Divisions. While in Division 1 working with the team is a full-time activity, coaches in Division 2, but foremost in Division 3, may have additional responsibilities such as teaching and mentoring (Grant et al., 2014). To obtain the NCAA recruiting certification, coaches in Divisions 1 and 2 need to pass an on-site test, while coaches in Division 3 have to take an online open book Rules Test (NCAA, 2017b). The passing grade is lower for coaches in Division 2 than for coaches in Division 1. Coaches in Division 3 do not have to pass the test. “A passing score is not a Division 3 requirement; however this feature will be used at the director of athletics discretion” (NCAA, 2017b). This means that while the pool of potential candidates is limited in Division 1 and 2, it is significantly larger in Division 3, a fact that might have a significant influence on who a University is going to hire.

Finally, in our dataset Division 3 (15.0%) has the highest share of Black students. In Division 1 (8.4%) and in Division 2 (13.3%) the share is statistically significantly higher. Additionally, the majority of Division 3 schools are private while the majority of Division 1 and 2 schools are public.

## 3. Data and Methods

### 3.1. Longitudinal and Spatial Analysis

To find a representative number of Black coaches and analyze the influence of the creation of the Division system on racial representation, we use data starting from 1947, when the Division system was first introduced.

**Figure 3:**
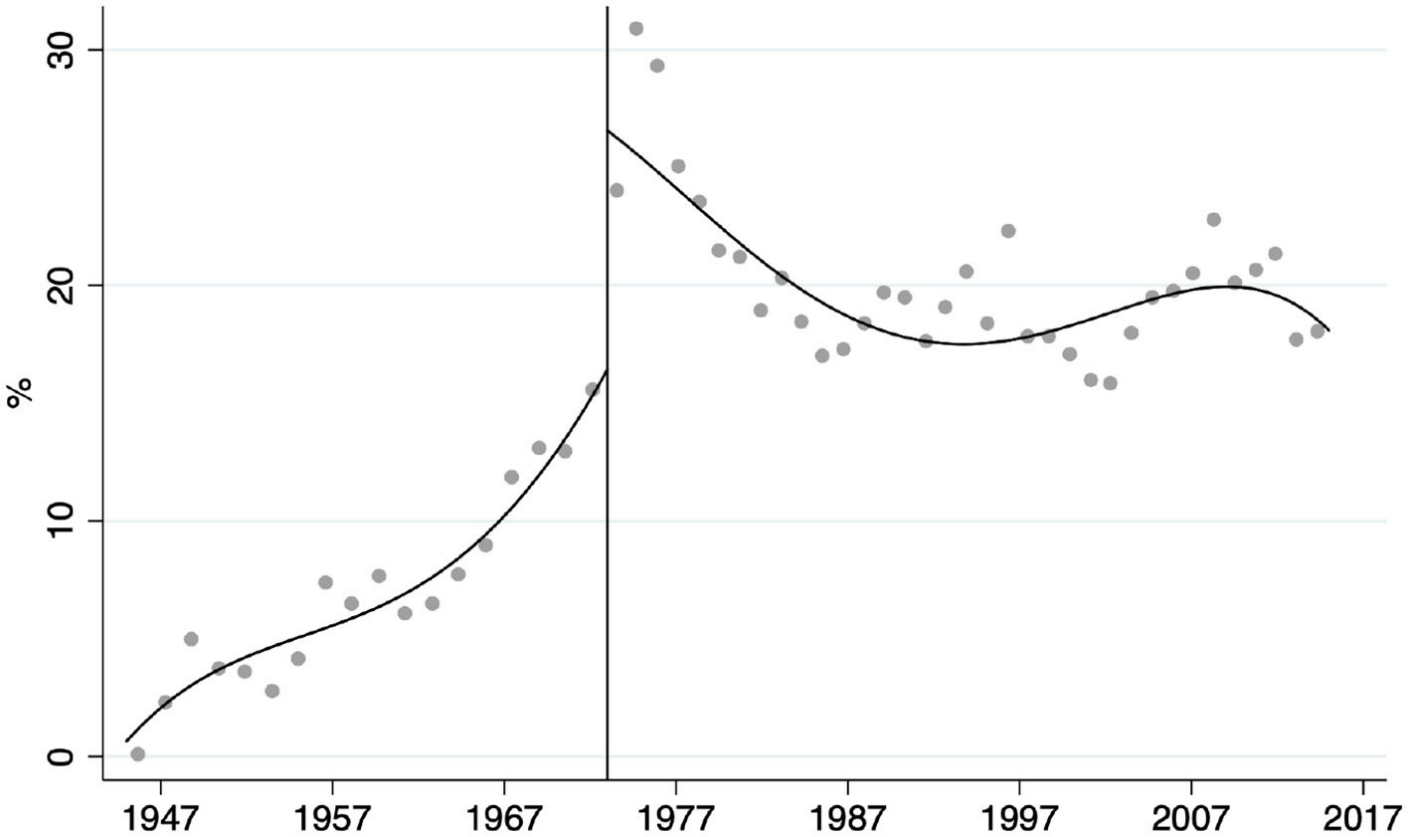
Black coaches in division 2.

Our aim is to assess what the split up meant for Black coaches in Division 2. Figure 3 shows how the share of Black coaches changed before and after the split in 1973 in Division 2. The point estimator for 1973, of the regression discontinuity design, using the conventional method is 10.6% (with a 95% confidence interval between 3.6 and 17.7%). After the split in 1973, the average share of Black coaches in Division 2 was significantly higher. In Table 1, we observe that the largest share of Division 3 teams in 1973 consists of former Division 2 teams.

**Figure 4:**
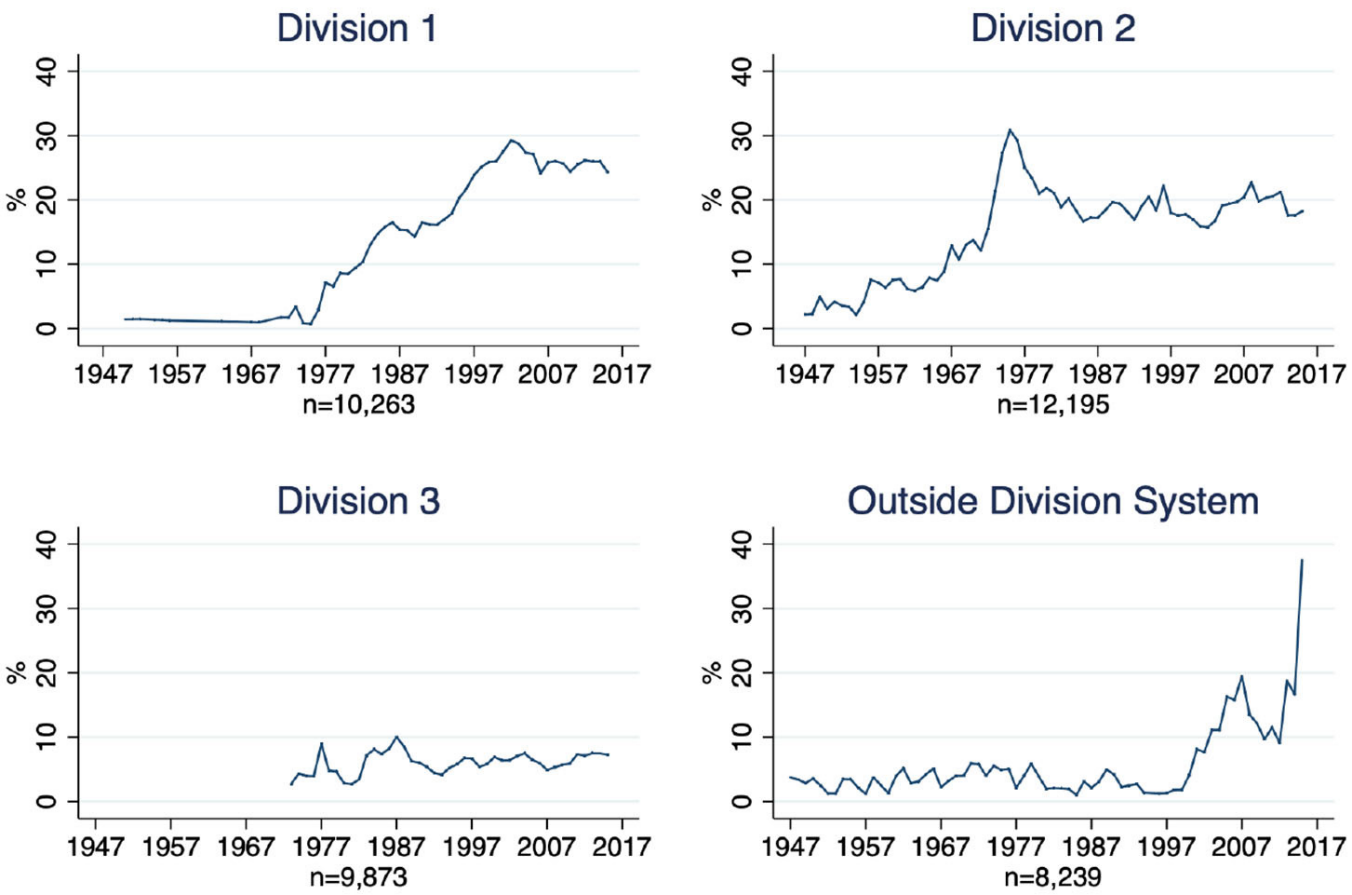
Black coaches in divisions.

In the next step we examine if the higher (lower) share of Black coaches in Division 2 (Division 3) was temporary. Therefore, we analyze how the share of Black coaches in Division 2 and 3 evolved. Figure 4 shows the share of Black coaches for all divisions. Since 1995, the share has been above 20% in Division 1, above 15% in Division 2, and below 10% in Division 3.

To summarize, Figures 3, 4 have a twofold purpose. First, Figure 4 shows that the share of Black coaches in Division 3 is significantly lower than in the other two divisions. Second, Figure 3 demonstrates that the 1973 split had an immediate impact on the share of coaches in Division 2.

Historical differences between southern and northern states regarding discriminatory behaviors and stereotypes are frequently discussed in the literature (Wilson, 1996; Kuklinski et al., 1997). Laws upholding racial segregation, effectively discriminating against Blacks, were still in place in several southern states until 1964 (Cole and Ring, 2012). Protests were often violently suppressed, e.g., sit-in movements, such as the Nashville sit-ins (Morris, 1981), and the freedom riders (Arsenault, 2007). Additionally, ongoing discussions regarding symbols of the confederacy preserve the image of a southern population prone to support racism (Wright and Esses, 2017). Thus, it is important to examine if the share of Black coaches is geographically clustered. The geographical examination is important to corroborate whether there are institutions from specific regions with a significantly different representation of Blacks among coaches^12^.

**Map 1:**
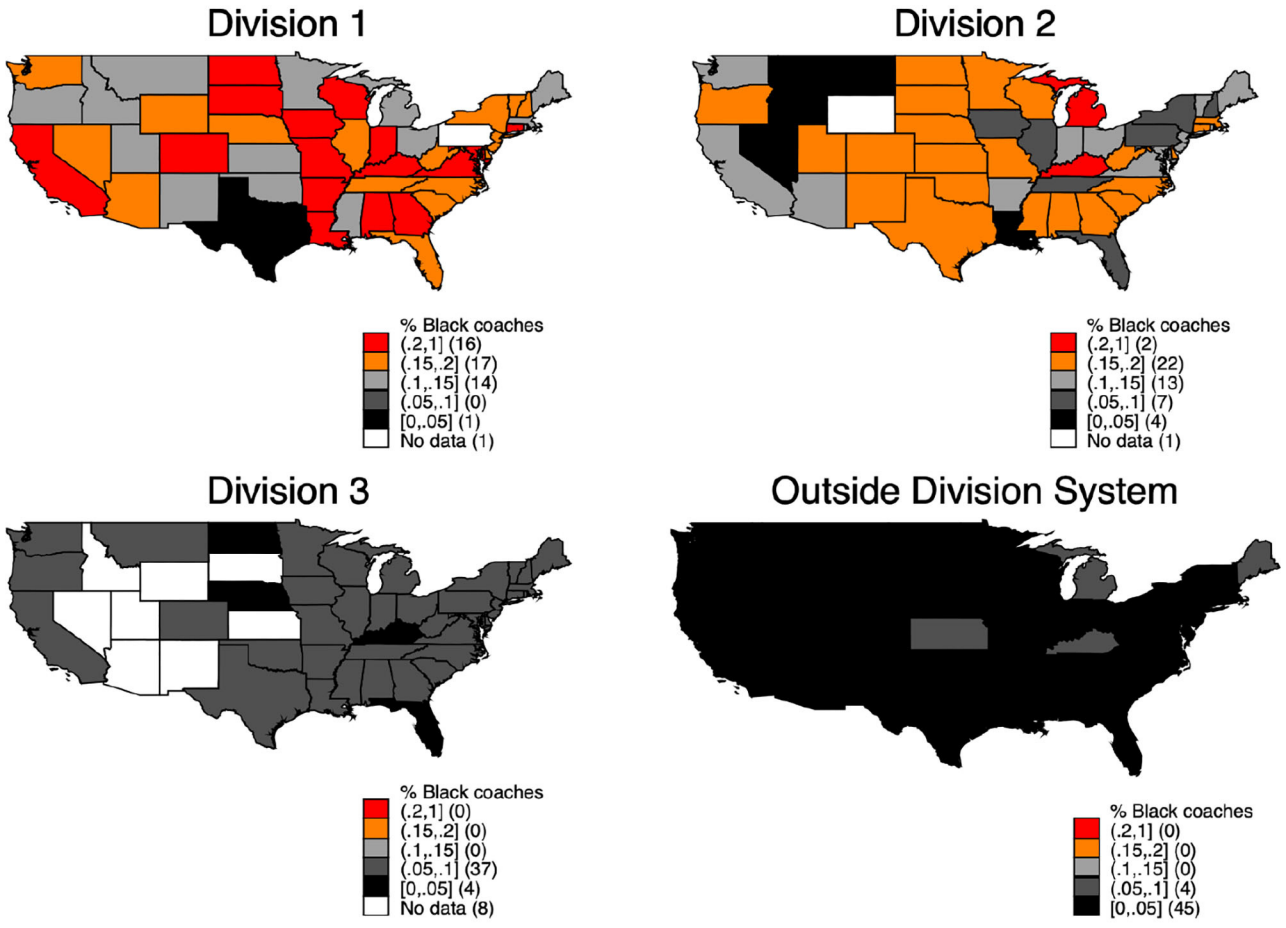
Coaches in divisions.

Map 1 shows the share of Black coaches in every division since 1973^13^. The map confirms the results from Figure 4, regarding the decrease in the share of Black coaches in Division 3 and outside the Division system. Additionally, we see that a lower share of Black coaches is not clustered in the southern states and a higher share is not clustered on either the east or the west coast. However, these results do not completely clarify whether the shares are spatially similar, since differences within a state can exist.

**Map 2:**
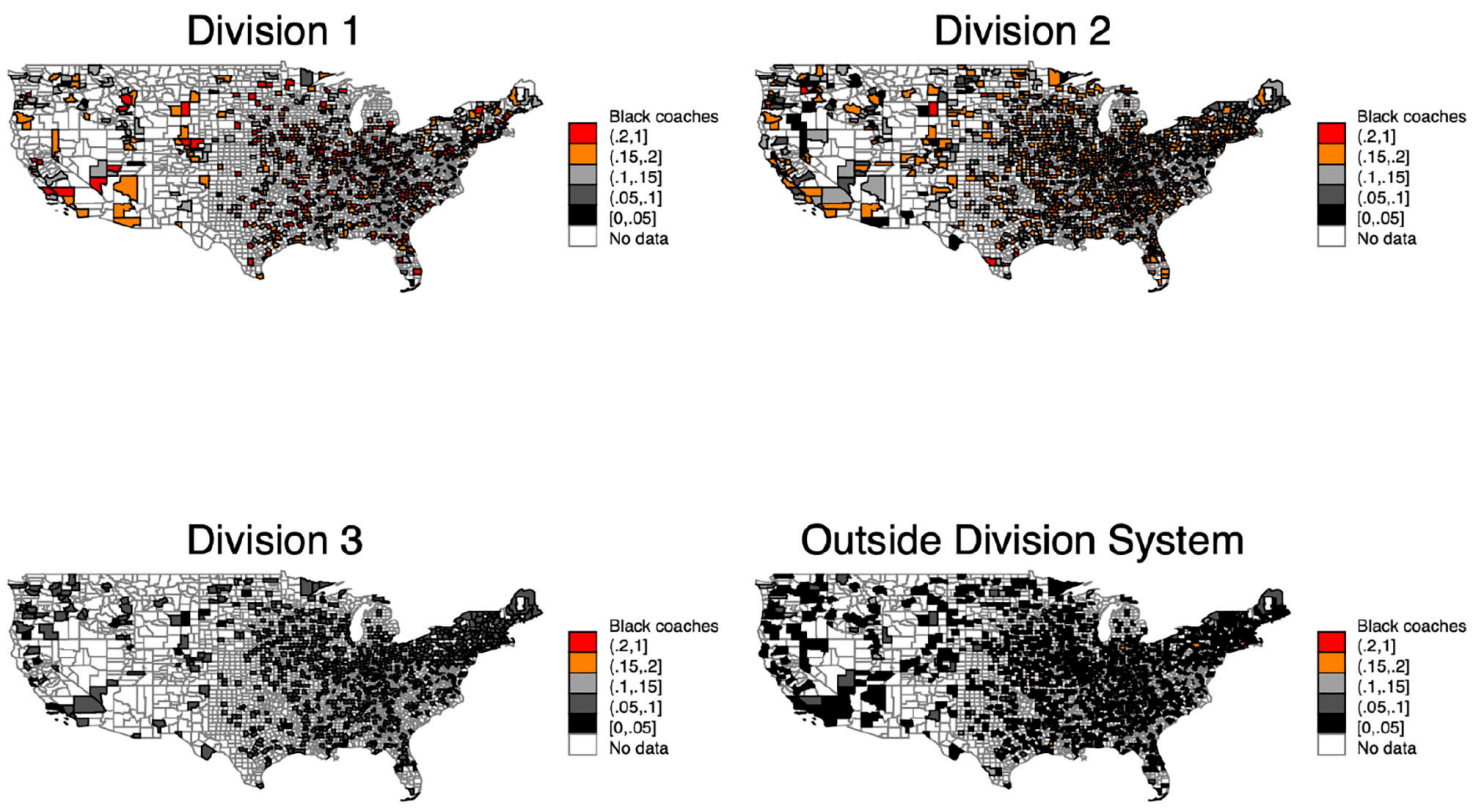
Coaches in divisions in counties.

Map 2 examines the share of Black coaches at the county level instead of at the state level (Map 1). We include the county level because regional differences between institutions within the same state might be responsible for the different share of Black coaches.

Map 2 clarifies one important point; the share of Black coaches looks independent of both county and region. While some counties have a comparably higher share of Black coaches in Division 1 and 2, the same counties have a lower share in Division 3 and outside the Division system. These results confirm that neither the states nor the regions are responsible for the different share of Black coaches.

### 3.2. Empirical Approach

Next, we analyze whether institutional characteristics help to explain the share of Black coaches. We construct the following model:

$$Y_{itd}=\beta_{0}+\zeta *X_{itd}+Y_{itd}+\gamma_{itd}+\epsilon_{itd}$$

The above equation specifies the model we use. The regressand is the race of the coach who is hired by school *i* at time *t* in Division *d*. *Y* is a binary regressand that distinguishes between African- and White coaches. ϵ is a random error term.

Table 2 provides an overview of the data. We distinguish between four different ethnic groups: African, Asian, Hispanic, and White. Asians and Hispanics compose <0.1% of the dataset. We therefore stick to the groups as Black and White^14^. Unfortunately, we do not have observations to include female coaches.

**Table 2:** Descriptive statistics for coaches.

**Variables**	**Mean**	**Std. Dev**.	**Min**.	**Max**.	***N***
Year	1984	19.3	1947	2015	43,250
White coaches	0.900	0.300	0	1	37,313
White coaches in Division 1	0.842	0.296	0	1	10,892
White coaches in Division 2	0.851	0.296	0	1	8,820
White coaches in Division 3	0.939	0.296	0	1	7,745
White coaches Outside Division System	0.977	0.296	0	1	9,856
HBCU	0.055	0.228	0	1	15,795
Private institution	0.559	0.496	0	1	18,440
Winning Percentage	51.1	19.6	0	100	43,250
Black students	0.101	0.181	0	1	18,297
Total enrolled students	10,729	22,697	67	272,128	18,297
Average school faculty salary*	52,913	18,569	0	166,697	16,210
Tuition and fees for students in US 2012 $**	8,984	8,627	0	45,212	18,113
Observations in Division 1	0.237	0.425	0	1	43,250
Observations in Division 2	0.233	0.423	0	1	43,250
Observations in Division 3	0.185	0.388	0	1	43,250
Observations Outside Division System	0.346	0.476	0	1	43,250
Total number of institutions				1,219	43,250
State***				56	43,250
County				544	43,250

* *In 0.4% of the cases did Institutions either pay no or a very low average school faculty salary (<1,000 $). For example, Wisconsin Lutheran (with a total enrollment of 203 students in 1988) or Corban (with a total enrollment of 288 students in 1988)*.

** *Institutions without tuition fees are Air Force Academy, Coast Guard Academy, Naval Academy, Merchant Marine Academy, and the Military (West Point) Academy*.

*** *Includes Canadian provinces and US territories*.

The vector **X** is a set of school characteristics. Previous studies find that the characteristics of schools can have an influence on the racial differences that we observe in the labor market. For example, Arcidiacono and Koedel (2014) find that urban campuses and low-quality schools explain the rate gap between Black and White students. Therefore, we incorporate the following characteristics:

At the institutional level, we indicate if the institution is a historically Black college or University (HBCU). Jones and Bell (2016) report that 55 HBCU are part of the NCAA: 23 in Division 1, 29 in Division 2, and three in Division 3. HBCUs “were established to serve the educational needs of Black Americans. Prior to the time of their establishment, and for many years afterwards, Blacks were generally denied admission to traditionally White institutions. As a result, HBCUs became the principle means for providing postsecondary education to Black Americans” (U.S. Department of Education, 1991). Accordingly, it is important to establish whether if Black coaches are mainly hired at HBCUs, as discussed in Fryer and Greenstone (2010) and LaFave et al. (2018). The average number of enrolled students, in our sample, is smaller for HBCUs than for other institutions (4,193 compared to 10,984). 67.52% of the HBCUs observations are public schools, 41.49% of none HBCUs are public schools. In addition to HBCUs, we include the share (percentage) of Black students to capture how different shares influence a school's decision to hire an Black coach.

The size of an institution, determined by the number of enrolled students, correlates to some extent only to the division in which the institution participates^15^. Several institutions with a large number of students have no athletic participation in either Division 1 and/or 2. Total enrollment, in our sample, for black students is the highest for Division 1. However, the share of Black students is the highest for Division 3. The same reasoning applies for school tuition. The analysis includes both the total number of enrolled students and the net student tuition, which is the amount of money the institution takes in from students after institutional grant aid is provided.

We differentiate whether an institution is public or private. Public institutions could have hiring regulations that differ from private institutions^16^. Additionally, more black students represent 10.7% of enrolled students at public schools and 8.1% at private schools. We include how much an institution pays on average to their faculty members. The choice of a new coach can significantly depend on the wage an institution offers. Moreover, this decision also depends on the team's records. Therefore, the analysis includes a one-year time lag of the school's winning percentage.

**Y** are year fixed effects. We cluster at the county level; γ. We distinguish between counties because historically, discrimination is unevenly distributed in the US^17^,^18^.

We gathered the data from three different sources. First, yearly college data (year, state, school, division, and winning percentage) was available at the NCAA homepage. The location was available on the institution's homepage. When missing, we looked the geographical information up in the Integrated Postsecondary Education Data System (IPEDS). Second, ethnic coach information for recent observations was available on the school's sports homepage. However, the majority of the observations was gathered by inspecting historical yearbook records. A direct contact with schools or coaches was needed for several missing observations. If yearbook records or school contacts were unable to provide us with this information we omitted the coach from the following analysis. Third, financial and enrollment information was extracted from the IPEDS.

We use data beginning with 1987 for the empirical analysis because complete IPEDS data is only available since then. The graphical analysis employs the whole dataset since the NCAA began the Division system.

To examine the difference *D* between the schools, the following formula is presented:

$$D_{it}=Y_{Ait}-Y_{Bit}$$

where *Y* is the regressand (that is, *CoachRace*) of the previously specified model. *A* specifies either Division 1 and Division 2 combined (models 1 and 2) or Division 2 (models 3 and 4). *B* specifies either Division 3 and schools outside the Division system (models 1 and 3) or Division 3 (models 2 and 4). We choose these four models because the split in 1973 affected all divisions and also schools from outside the Division system (see, Table 2).

To examine *D* we use the Blinder-Oaxaca decomposition. This method is often used to assess group differences (Angrist et al., 2013).

Table 3 shows the results from the decomposition. In every model the share of Black coaches is significantly higher in Division 1 and Division 2. The magnitude of the difference is lower in model 3 and 4. The decomposition divides the difference in statistical unexplained and explained variation. Both explained and unexplained variation consist of the covariates of our model.

**Table 3:** Model results (OLS).

**Variables**	**Blinder-Oaxaca dependent variable Black or White Coach (0/1)**							
	**Model 1**		**Model 2**		**Model 3**		**Model 4**	
	**Div. 1 & 2**	**Div. 3 & No**	**Div. 1 & 2**	**Div. 3**	**Div. 2**	**Div. 3 & No**	**Div. 2**	**Div. 3**
Observations	7,945	5,615	7,945	4,498	3,206	5,615	3,206	4,498
*CoachRace*	0.789***	0.943***	0.789***	0.941***	0.814***	0.943***	0.814***	0.941***
	(0.015)	(0.010)	(0.015)	(0.011)	(0.025)	(0.010)	(0.025)	(0.011)
*D*	0.154***		0.153***		0.129***		0.127***	
	(0.017)		(0.018)		(0.027)		(0.027)	
Explained	0.079***		0.074***		0.071***		0.067***	
	(0.014)		(0.014)		(0.022)		(0.023)	
Unexplained	0.075***		0.078***		0.057**		0.060**	
	(0.017)		(0.018)		(0.022)		(0.024)	
	**Expl**.	**Unexpl**.	**Expl**.	**Unexpl**.	**Expl**.	**Unexpl**.	**Expl**.	**Unexpl**.
HBCU	0.036***	0.028	0.039***	0.163	0.056***	−0.149	0.059***	−0.014
	(0.008)	(0.351)	(0.008)	(0.460)	(0.017)	(0.328)	(0.017)	(0.470)
Total enrolled students	0.002	0.005	0.002	0.005	0.000	−0.005	−0.000	−0.005
	(0.004)	(0.015)	(0.003)	(0.016)	(0.000)	(0.007)	(0.000)	(0.008)
Share of Black students	0.025***	−0.002	0.026***	−0.000	0.029***	−0.013	0.029***	−0.011
	(0.007)	(0.014)	(0.007)	(0.017)	(0.011)	(0.016)	(0.011)	(0.020)
Last years winning percentage	−0.001	−0.016	−0.001	−0.015	−0.002	0.026	−0.002*	0.028
	(0.001)	(0.022)	(0.001)	(0.025)	(0.001)	(0.027)	(0.001)	(0.030)
Net student tuition	0.004	0.004	0.003	0.006	−0.001	0.001	−0.001	0.003
	(0.003)	(0.015)	(0.002)	(0.016)	(0.001)	(0.017)	(0.002)	(0.017)
Public institution	0.009	−0.006	−0.004	0.007	0.007	−0.010	0.007	−0.008
	(0.006)	(0.021)	(0.007)	(0.027)	(0.007)	(0.025)	(0.007)	(0.030)
Average school faculty salary	0.014***	0.037	0.004	0.019	−0.003	−0.037	−0.013**	−0.057
	(0.005)	(0.087)	(0.003)	(0.100)	(0.003)	(0.141)	(0.006)	(0.151)
Constant		−0.046		−0.188		0.110		−0.032
		(0.351)		(0.462)		(0.344)		(0.495)
Observations	13,560		12,443		8,821		7,704	
Year FE	Y		Y		Y		Y	
Cluster County Level	Y		Y		Y		Y	

Robust standard errors in parentheses. ***p < 0.01, **p < 0.05, *p < 0.1.

A large share of the explainable differences in every model can be explained by the fact that Division 3 institutions and institutions outside the Division system are less often HBCUs and have a lower share of Black students. All other covariates have either no statistically significant influence or their magnitude varies throughout the models (for example, average school faculty salary). Accordingly, the share of Black coaches in Division 3 and outside the Division system would be between 6.7 and 7.9% higher if these institutions worked under similar conditions as institutions in Division 1 and 2.

Nonetheless, the magnitude of the unexplainable part, which in the literature is often associated with discrimination (as it cannot be explained by any of the covariates, cf. Jann, 2008; Le and Nguyen, 2018), is substantial and highly statistically significant. It ranges between 5.7% (model 3) and 7.8% (model 2).

## 4. Robustness Checks

The analysis includes the following robustness checks:

In Figure 3, our point estimate in 1973 is estimated by using the conventional method in regression discontinuity design. However, recent empirical literature (e.g., Calonico et al., 2015) suggests that other estimators are also appropriate. Accordingly, we include the so-called bias-corrected and robust estimators in Table 4. All estimators are larger than 9.8% and have a 95% confidence interval which, at the lowest, is above zero.

**Table 4:** Point estimation results for Figure 3.

**Variables**	**RD estimate**	**Confidence interval**
Conventional	0.106***	[0.036;0.177]
	(0.0359)	
Bias-corrected	0.0981***	[0.028;0.168]
	(0.0359)	
Robust	0.0981**	[0.016;0.180]
	(0.0419)	
Observations	8,977	

*Standard errors in parentheses*.

***p < 0.01, **p < 0.05, *p < 0.1.

The analysis from Table 3 can also be performed with a logit. For a more detailed discussion regarding the benefit of using either model see both Angrist (2001) and Beck and Katz (2011). However, the differences between the logit model (Table 5) and the OLS model (Table 3) are only marginal. The most important difference between the two models is that the unexplained part for model 1 and model 3 is larger in the logit model (Table 5).

**Table 5:** Model Results (Logit).

**Variables**	**Blinder-Oaxaca dependent variable Black or White Coach (0/1)**							
	**Model 1**		**Model 2**		**Model 3**		**Model 4**	
	**Div. 1 & 2**	**Div. 3 & No**	**Div. 1 & 2**	**Div. 3**	**Div. 2**	**Div. 3 & No**	**Div. 2**	**Div. 3**
Observations	7,945	5,615	7,945	4,477	3,206	5,615	3,206	4,477
*CoachRace*	0.789***	0.943***	0.789***	0.946***	0.814***	0.943***	0.814***	0.946***
	(0.013)	(0.009)	(0.013)	(0.011)	(0.009)	(0.020)	(0.011)	(0.019)
*D*	0.154***		0.157***		0.129***		0.131***	
	(0.017)		(0.018)		(0.021)		(0.022)	
Explained	0.066***		0.069***		0.070***		0.072***	
	(0.008)		(0.008)		(0.010)		(0.011)	
Unexplained	0.088***		0.089***		0.058**		0.059**	
	(0.018)		(0.019)		(0.021)		(0.022)	
	**Expl**.	**Unexpl**.	**Expl**.	**Unexpl**.	**Expl**.	**Unexpl**.	**Expl**.	**Unexpl**.
HBCU	0.021***	−0.096	0.027***	−0.442***	0.046***	−0.116	0.059***	−0.371***
	(0.005)	(0.173)	(0.006)	(0.084)	(0.015)	(0.122)	(0.022)	(0.087)
Total enrolled students	0.002	0.004	0.002	0.005	0.000	−0.005	−0.000	−0.000
	(0.003)	(0.007)	(0.003)	(0.008)	(0.000)	(0.003)	(0.000)	(0.004)
Share of black students	0.024***	−0.017	0.029***	−0.017	0.050***	−0.016*	0.061***	−0.016
	(0.005)	(0.011)	(0.006)	(0.015)	(0.013)	(0.010)	(0.018)	(0.012)
Last years winning percentage	−0.001	0.043*	−0.002	0.034	−0.004	0.045	−0.006	0.039
	(0.001)	(0.024)	(0.002)	(0.027)	(0.003)	(0.022)	(0.004)	(0.024)
Net student tuition	0.002	−0.000	0.002	0.000	−0.001	0.000	−0.002	0.000
	(0.002)	(0.006)	(0.002)	(0.007)	(0.002)	(0.007)	(0.003)	(0.007)
Public institution	0.003	0.063	0.003	0.074	−0.008	0.084	−0.011	0.093
	(0.009)	(0.061)	(0.011)	(0.069)	(0.019)	(0.057)	(0.025)	(0.063)
Average school faculty salary	0.016***	−0.066	0.005	−0.079	−0.007	−0.068	−0.030**	−0.077
	(0.005)	(0.071)	(0.004)	(0.077)	(0.006)	(0.077)	(0.018)	(0.081)
Constant		0.121		0.454***		0.106		0.351**
		(0.160)		(0.118)		(0.120)		(0.116)
Observations	13,560		12,422		8,821		7,683	
Year FE	Y		Y		Y		Y	
Cluster county level	Y		Y		Y		Y	

Robust standard errors in parentheses. ***p < 0.01, **p < 0.05, *p < 0.1.

Additionally, the covariates we use in the model might distort the results as several coaches and years are not included when adjusting for additional variables. Thus, a full sample with all observations might show a different picture. Therefore, we include Table 6 without covariates.

**Table 6:** Model results.

**Variables**	**Blinder-Oaxaca dependent variable Black or White Coach (0/1)**							
	**Model 1**		**Model 2**		**Model 3**		**Model 4**	
	**Div. 1 & 2**	**Div. 3 & No**	**Div. 1 & 2**	**Div. 3**	**Div. 2**	**Div. 3 & No**	**Div. 2**	**Div. 3**
Observations	19,945	17,877	19,945	7,881	8,977	17,877	8,977	7,881
*CoachRace*	0.843***	0.958***	0.843***	0.938***	0.846***	0.959***	0.846***	0.938***
	(0.003)	(0.001)	(0.003)	(0.003)	(0.003)	(0.001)	(0.004)	(0.003)
*D*	0.115***		0.095***		0.113***		0.092***	
	(0.003)		(0.004)		(0.004)		(0.005)	
Constant	0.115***		0.095***		0.113***		0.092***	
		(0.003)		(0.004)		(0.004)		(0.005)
Observations	37,822		27,826		26,854		16,858	

Robust standard errors in parentheses. ***p < 0.01, **p < 0.05, *p < 0.1.

**Map 3:**
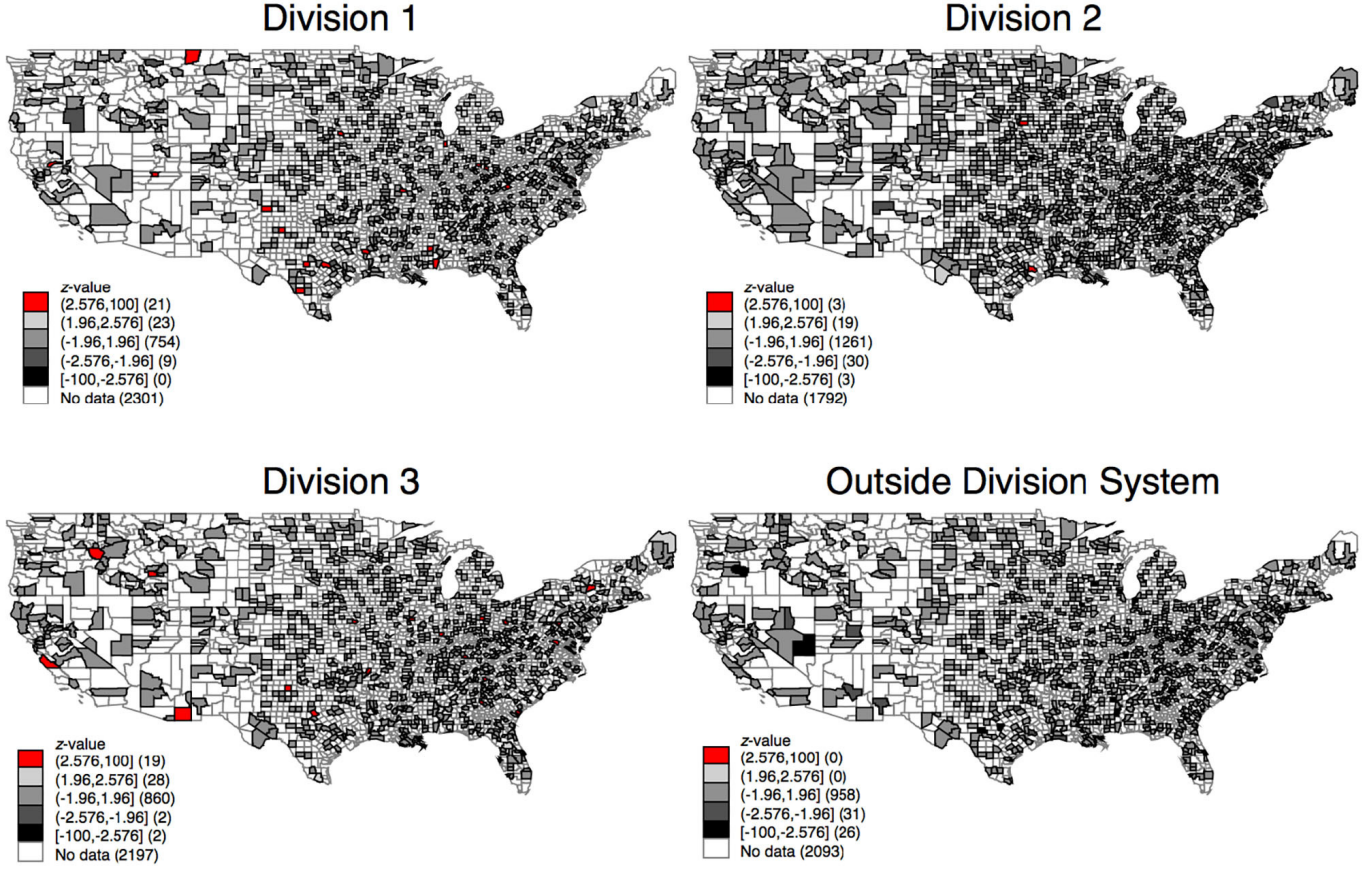
Z-scores for coaches in divisions in counties.

We examine the data from Map 2 using a hot- and cold-spot analysis. We use the getis-ord analysis to examine if a county and counties in its vicinity comprise a regional cluster. It examines whether one region has either a high or low value and if the regions in its vicinity also have a high or low value. Our results show that no clustered regions throughout the US exist in relation to the results of Map 3. For discrimination on the county level to be valid, hot spots should be at the same spots throughout the maps over time.

## 5. Discussion

Arrow (1998) explains that discrimination was omnipresent in daily life in the US before the Civil Rights Act of 1964 (for example, segregation in public facilities or exclusion from good jobs) and thus there was no need to detect it in the labor setting. This argument is correct when observing the participation of Black athletes or coaches in the beginning of college basketball (Yetman et al., 1982). Additionally, Stevenson (2010) shows that the inclusion of female athletes had a significant positive impact on female college participation. However, no legal basis for active discriminatory behavior exists any longer. Accordingly, the asymmetric representation of Black players and coaches, which is represented in Figure 1, denotes an intriguing but irritating issue.

This study shows a historical under-representation of Black coaches in NCAA college basketball that still persists today and points toward several factors that have an influence. The results from this study show that the number of Black coaches is significantly lower in Division 3 than in the other Divisions. Differences in the importance and visibility of performance among divisions may explain the higher representation of Black coaches in Divisions 1 and 2. Gordon (2008) states that the issue of under-representation of Blacks in leadership positions in college sports poses three main problems: first, the lack of fairness and meritocracy that sports are supposed to represent; second, the lack of mentors and role models for Black students; and, third, the influential position of White head coaches within the organization.

The results also show that Black coaches do not work in geographically clustered regions. Thus, they are not clustered in specific cities, counties, or states. This result is novel to the literature on race and employment of coaches in college sports, which overlooks the geographical distribution of the differences. Our results do not support the historical and problematic connection between Black citizens and several states. The under-representation of Black coaches is embedded in the US. Additionally, while several differences within states exists (e.g., ethnic or wealth distribution), none of them are responsible for this under-representation.

The motivation for racial preferences and the reasons for manifestations of discrimination are diverse, which prevents any theoretical reasoning from identifying definitive causes and effects (Arrow, 1998). The results from our study empirically show the characteristics of colleges and Universities and subtleties about the Division system of NCAA Men's Basketball which explain part of the under-representation of Black coaches.

Previous experience is a valuable asset in management and leadership positions in sports teams (Dawson and Dobson, 2002), and specifically in basketball in the US (Goodall et al., 2011). However, the results in this study show that Black coaches represent only 16.4% of all coaches in NCAA Men's Basketball. This is unreasonable because most coaches in NCAA basketball previously played in the NCAA and, thus, have knowledge and experience in the same industry. For example, in 2020, 18 of the 20 highest-ranked Division 1 teams had a coach who was a former player^19^. Accordingly, the pool of potential candidates consists of at least 60% Blacks. If athletic departments deliberately neglect Black coaches in the hiring process, not only Blacks are compromised but also the performance of the institutions.

A possible cause of this disparity is the network approach, according to which the maintenance of social interactions and network referrals perpetuates discriminatory behaviors. Research shows that social networks significantly differ depending on the race and gender of the individual and, in turn, might restrain the individual's possibilities (Ibarra, 1993, 1995; Day, 2015, 2018). Hawkins (2013) and Regan (2014) argue that NCAA institutions are predominantly managed by Whites, which might affect the hiring process. Racial bias in sports institutions acts in accordance with the glass ceiling phenomenon, which describes how invisible barriers prevent certain minority groups from reaching top leadership positions (Morrison et al., 1987). Additionally, because of the permanently low number of Black coaches in college sports, the size of the respective network of Black coaches is small and a hindrance for them (Day and McDonald, 2010). Two covariates diminish this effect in college basketball: the number of enrolled Black students and the division in which the institutions compete.

The decomposition amplifies findings that the number of enrolled Black students in colleges and Universities has a positive influence on the representation of Black coaches. The preference of Black students for coaches with a similar racial background diminishes the representation gap. These results are in line with those of Savage and Seebruck (2016), who find that subordinates and supervisors from the same race in intercollegiate athletic departments are more likely to support each other.

Research highlights the positive impact of public Universities on the participation rates of racial minorities over the past decades (Duderstadt and Womack, 2003; Strayhorn, 2017). However, research also argues that active participation and academic success do not only depend on the public status of a University, but on social involvement factors such as support networks and connections to the community, and campus racial composition (Allen, 1992; Strayhorn, 2017). In this line, our results show that neither public nor private institutions have a significant influence on the representation of Black coaches. However, the variables capturing the share of Black students and HBCUs have a strong influence in our model. This influence might capture the effect of public institutions as the majority of HBCUs in our sample are public.

Most Black coaches are employed in colleges and Universities in Division 1 and Division 2 (see Figure 4). This result does not necessarily have negative economic implications for Black coaches, since these jobs are arguably superior to jobs in the lower division. However, the result does compromise the number of role models from racial minorities and the access to influential networks within the institutions. Figure 4 still reveals a disproportionate share of Black coaches in Division 1 and 2, if we consider the number of Black players. Moreover, the lower representation of Black coaches in a less competitive setting (Division 3) has important implications for research on racial discrimination.

The notion of White “minds” managing Blacks is reminiscent of a burden perpetuated in the colonial model that has determined the role of Blacks in US society and attracted the attention of academics (e.g., Hawkins, 2013), and broader audiences (e.g., Coates, 2015). The uneven distribution of Black coaches throughout the divisions has been exacerbated with the introduction of Division 3. One explanation is that the stronger regulations in Division 1 and 2, and the need to deliver performance, hinders discrimination. This supports the argument that discrimination based on prejudices is less prominent in highly competitive or norm-based environments than in more ambiguous scenarios (Dovidio and Gaertner, 2000).

Another possible explanation for the under-representation of Black coaches in Division 3 is related to the idea of imperfect information (Darity and Mason, 1998). Most of the athletic departments are managed by Whites. Because it is not critical to hire the best possible candidate, due to the ambiguous requirements regarding performance delivery, coaches who are a priori culturally closer are favorites. Along these lines, Giuliano et al. (2009) analyze the new hires of more than 700 retail stores in the US in the late 90's, and find that non-Black managers are more likely to hire White employees than Black managers, especially in the South. This finding differs from our results, as we do not find significant differences in the representation of Black coaches across states.

The requirements for coaches can also help to explain the difference in the number of Black coaches across divisions. The requirements refer to the requisite qualification to become a coach in the different divisions. The test for coaches in Division 3 is a formality and opens the market for a broad set of candidates. Division 1 and 2 candidates must pass a sophisticated test controlled by the NCAA. Moreover, the need to hire a coach who performs well limits the number of possible White coaches. Fewer Black coaches are hired in Division 3 because they compete with a larger share of White candidates.

The introduction of Division 3 in 1973 shaped the role of Black coaches in NCAA Men's Basketball. The structural change aimed to enhance competitive balance within the divisions by reducing the differences between big and small institutions within a Division, but the policy change also affected the position of Black coaches in the labor market. Prior research notes similar unintended influences of policies in the representation of coaches from minorities.

Title IX of the Education Amendments was expected to improve the overall role of women in college sports. However, the changes resulted in a decrease in the proportion of women coaches (Acosta and Carpenter, 2000). Research offers a number of explanations that relate to more pervasive job opportunities, homologous reproduction, perceived competence, and satisfaction (Stangl and Kane, 1991; Sagas and Batista, 2001; Cunningham and Sagas, 2002; Kilty, 2006). Some of these factors may also explain the under-representation of Black coaches in Division 3 that followed its creation.

To counteract, the Strategic Alliance Matching Grant and the Coaching Enhancement Grant Program for institutions in Division 2 and 3 aimed to promote coaching positions for ethnic minorities (Lapchick et al., 2011; NCAA, 2017c). Despite this effort, we find that the number of Black coaches remains significantly lower. This result sets the ground for future research on race and employment to examine meso-level factors and individual factors that could have indirectly shaped the role of racial minorities, especially in the context of college sports (Cunningham, 2020).

This result also calls for complementary actions that can increase the access of Blacks to head coaching positions. These would include measures such as training programs focused on Black coaches (Demers, 2009), ensuring transparency in the hiring process (Van den Brink et al., 2010), and setting racial quotas for academic personnel (McCrary, 2007). Additionally, selection criteria for coaches are rarely publicly accessible in the NCAA: schools and athletics committees have the final say in the hiring process. Because competition results, school preferences, and organizational structure can play a role in this process, policies based on transparency have the potential to improve the representation of coaches from racial minorities. Such measures are especially interesting for future research as they would provide suggestions for possible improvement.

The supply side can also influence the results (McDowell et al., 2009). Previous studies show that the career path of Blacks and Whites differ in numerous respects (Falconer and Hays, 2006; Owens et al., 2010). Beyond organizational structure or institutionalized racism, micro-level factors such as self-imitating behaviors or capital investment can also harm the representation of minorities in leadership positions (Cunningham, 2020). Research finds that micro-level factors related to social capital deficiencies and worker's preferences, perceptions, and aspirations play a negative role in the representation of Blacks in intercollegiate leadership positions (Cunningham et al., 2001; McDowell et al., 2009; Cunningham and Singer, 2010; Day and McDonald, 2010; Steward and Cunningham, 2015).

Thus, available coaching positions in Division 3 might not receive very many applications due to lack of competitiveness and visibility. Additionally, research shows that Division 3 schools have fewer Black athletes than Division 1 or 2 (Lapchick et al., 2019). Thus, if schools want to hire a coach with insightful experience in the specific Division (viz., Division 3), fewer former Black athletes are available compared to Divisions 1 and 2. However, previous research also finds that not all racial minorities' capital deficiencies (e.g., qualifications or experiences) contribute to the low representation in sport leadership positions (McDowell and Cunningham, 2007). More research is needed in the field to disentangle these relationships.

Our results also have implications for other institutions that are concerned with the inclusion of minorities in executive positions. Coaches and other athletic staff members act as role models, who also provide support and are able to influence the career of Black students as in community colleges (Horton, 2015). Organizational bodies and supporting associations should take action to promote minorities in leadership positions (e.g., specialized training, quotas, transparency). In the specific college basketball context, the Black Coaches Association (BCA), which works to improve the conditions of coaches in the NCAA and the NCAA Men's Coaches Academy (NCAA, 2017c), can focus on divisions where the gap between Black and White coaches is larger. Future efforts must explore the differences in the structure and governance of the Divisions that generate racial inequality.

### 5.1. Limitations

This study has some limitations that prevent us from drawing more definitive conclusions. While we focused on men's college basketball, 1947–2015, data on women's college basketball is also available starting in 1981. Research shows that the share of Black coaches in women's basketball is comparably low and that discrimination is also a hindrance to career development for this group (cf., Borland and Bruening, 2010; Walker and Bopp, 2010; Gurney et al., 2017; Lapchick et al., 2019). Future research is needed to examine the situation in more detail, e.g., trends in representation and differences by Division.

We inferred the race of coaches based on available pictures. We assume that the simplistic categorization of Black and White coaches neglects some important features of self-identification and personal identity. In this line, when the skin color did not allow for a straightforward categorization, we automatically identified the coach as Black (<0.5% of all Black coaches were categorized in this way). If using a database with more recent observations, future research could incorporate self-reported measures of racial identity, e.g., from survey data (Lapchick et al., 2011).

The current study also lacks information on the demographics of the other agents in college sports. For example, we do not know the exact share of Black athletes in Divisions 1, 2, and 3, and, consequently, we are unable to show the actual Black coach-athlete ratio. In this line, Harper (2016) shows the graduation rate gap between Black and White students at Division 1 schools. Future research could use the results from the present study, i.e., the number of Black coaches, and compare it with the number of Black athletes. Future research could also control for the ethnic demographics of faculty, staff, and athletic department members. As this information was not available for all schools we were unable to control for these factors, which may well have an influence on the representation of racial minorities in executive leadership.

## 6. Conclusion

In this study, we used college basketball to examine how the number of Blacks and Whites evolved in the executive labor market since 1947. The results show that, although Blacks provide the large majority of potential coaches, their employment number has been below 20% and is not increasing. We found that Blacks are less often employed in Division 3. The highest share of Blacks is in Division 1 followed by Division 2. Our results clarify that this difference is not due to idiosyncratic institutional or geographical characteristics. Clearly, it is not the goal of the college basketball institutional bodies to increase discrimination:

*As a core value, the NCAA believes in and is committed to diversity, inclusion and gender equity among its student-athletes, coaches and administrators. We seek to establish and maintain an inclusive culture that fosters equitable participation for student-athletes and career opportunities for coaches and administrators from diverse backgrounds^20^*.

We provided several potential explanations for the lower number of Black coaches in college basketball. Career plan differences, lack of role models, characteristics of networks, hiring practices, and organizational structure and culture are all plausible causes. This study focuses on a non-diversity-related policy in the Division system—namely the creation of Division 3 in 1973—which created an imbalance of representation for Black coaches that still persists today. More research is needed to clarify the impact of each hindrance and examine the influence of policies on racial diversity in executive positions.

## Data Availability Statement

The data supporting the findings of this study are available at https://doi.org/10.7910/DVN/G3ST4O.

## Author Contributions

CN, CG-G, HD, and JC conceived the research idea, designed the experiment, performed the analyses, and wrote the manuscript. All authors contributed to the article and approved the submitted version.

## Conflict of Interest

The authors declare that the research was conducted in the absence of any commercial or financial relationships that could be construed as a potential conflict of interest.

## Footnotes

^1^We use the term “Black” as not all “Black” coaches might self-identify with the term African American.

^2^Less than 1% of Fortune 500 CEOs are of Black descent.

^3^This is astounding, as all five Nobel laureates working for the University do not earn as much. However, Berkeley is far from the exception, but is, rather, the standard for US colleges and Universities. For more information regarding the employees wages, see https://ucannualwage.ucop.edu/wage/.

^4^Additionally, research shows that Black coaches are also underrepresented in other programs, e.g., college women's soccer (Nesseler et al., 2020).

^5^One famous exception was Jackie Robinson who later became a professional baseball player.

^6^Elite-8 teams are the teams that compete in the quarterfinals of the largest NCAA Division 1 tournament every year. Unfortunately, no information is available regarding racial distribution for the complete division.

^7^A similar Division system with two or three divisions is in place for most other college sports.

^8^Table 1 from 1972 to 1973, is not a zero sum table because colleges and Universities also ceased participating or records are missing. Map 4 in the Appendix shows where the colleges and Universities that switched divisions were located. Note: We do not show colleges that did not participate in 1972.

^9^Data regarding division affiliation is not available for every college since 1947, thus, un-ranked colleges and missing data are the same in our analysis.

^10^In 2017, the maximum number of scholarships in Division 1 Basketball per college or University was 13, and limited to 9 in Division 2. Athletic departments can give partial scholarships (for instance, a 50% scholarship).

^11^For detailed information regarding the requirements to become an athlete in each division, please see NCAA (2017a).

^12^Although information about very few Canadian and Puerto Rican institutions exist, we do not include them in our analysis as they do not provide sufficient observations.

^13^We include the share of Black coaches for every decade in every division in the Appendix, compare Maps 5.1–5.4

^14^Changing the dataset to only Whites and Blacks does not change the results.

^15^For example, the correlation between enrolled students and Division 1 is 0.196.

^16^It is also reasonable to focus exclusively on undergraduate education (4-Year) and whether an institution is non-profit. However, this data was only available for a smaller dataset. We therefore decided not to include it.

^17^The following territories are included in the variable “State” but omitted in the analysis: British Columbia, Nova Scotia, Puerto Rico, and Quebec.

^18^Using either state, school, or county fixed effects does not alter the results.

^19^We were unable to detect if two coaches were former players.

^20^Extracted from NCAA (2017d).
