# Supplementary material for: Race and Employment: The Historical Case of Head Coaches in College Basketball
Source: Front Sociol. 2020 Oct 8;5:69. doi: 10.3389/fsoc.2020.00069 (PMC8022560; doi:10.3389/fsoc.2020.00069)
Supplement: Supplementary file 1 [file Data_Sheet_1.PDF]

TABLE 7

Institutions that Switched from Division 2 to 3 in 1973

School	State	School	State	School	State
Albion	MI	Hope	MI	Rensselaer	NY
Alfred	NY	Husson	ME	Rochester	NY
Alma	MI	Ithaca	NY	Rockford	IL
Aquinas	MI	Johnson St.	VT	Roger Williams	RI
Augustana	IL	Knox	IL	Rowan	NJ
Baldwin Wallace	OH	Kutztown	PA	SUNY Geneseo	NY
Beloit	WI	Lake Superior St.	MI	SUNY Oneonta	NY
Benedictine	IL	LeMoyne-Owen	TN	Saint Leo	FL
Binghamton	NY	Lincoln	PA	Salem St.	MA
Brandeis	MA	Luther	IA	Savannah St.	GA
Bridgewater	VA	Lycoming	PA	Southampton	NY
Brockport	NY	Macalester	MN	St. Lawrence	NY
CCNY	NY	Marist	NY	St. Olaf	MN
Capital	OH	McDaniel	MD	Stevens	NJ
Castleton	VT	Me.-Presque Isle	ME	Stillman	AL
Centre	KY	Memphis	TN	Suffolk	MA
Clark	MA	Millersville	PA	Susquehanna	PA
Clark Atlanta	GA	Millsaps	MS	Swarthmore	PA
Colorado Col.	CO	Monmouth	NJ	TCNJ	NJ
Colorado Mines	CO	Monmouth	IL	Thiel	PA
DePauw	IN	Montclair St.	NJ	Wartburg	IA
Defiance	OH	Morehouse	GA	Wash. & Jeff.	PA
Drury	MO	Mount Union	OH	Wash. & Lee	VA
Eckerd	FL	Muhlenberg	PA	Washington Col.	MD
Emory & Henry	VA	Neb. Wesleyan	NE	Westmont	CA
Findlay	OH	New Hampshire	NH	Wheaton	IL
Fitchburg St.	MA	Nichols	MA	Wiley	TX
Florida Tech	FL	North Park	IL	Wilkes	PA
Framingham St.	MA	Oberlin	OH	William Jewell	MO
Grinnell	IA	Ohio Northern	OH	Wittenberg	OH
Grove City	PA	Olivet	MI	Wm. Paterson	NJ
Hartwick	NY	Plattsburgh St.	NY	Worcester St.	MA
Hawthorne	CA	Pomona-Pitzer	CA	Yeshiva	NY
Heidelberg	OH	Pratt	NY		
Hiram	OH	Queens	NY		

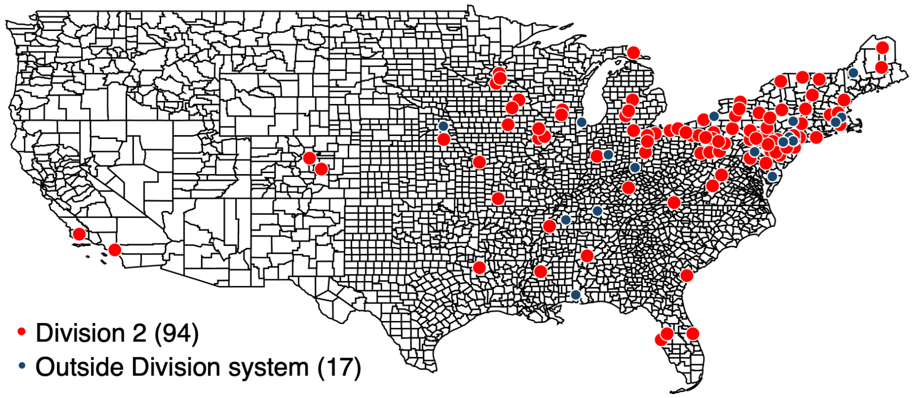

Map 4. Colleges and Universities
that switched to Division 3 in 1973

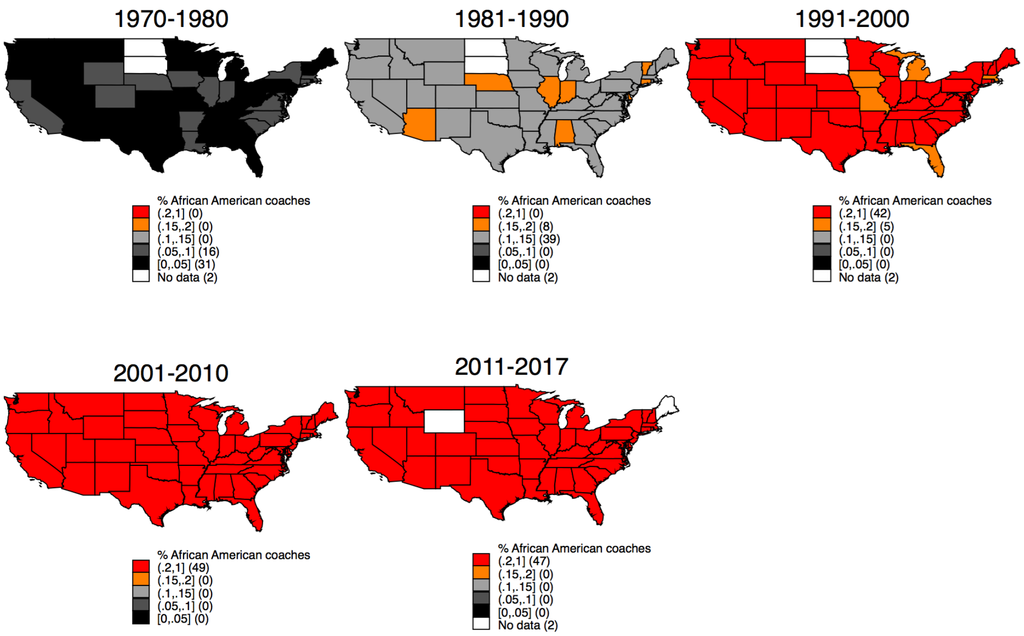

Map 5.1. Coaches in Division 1

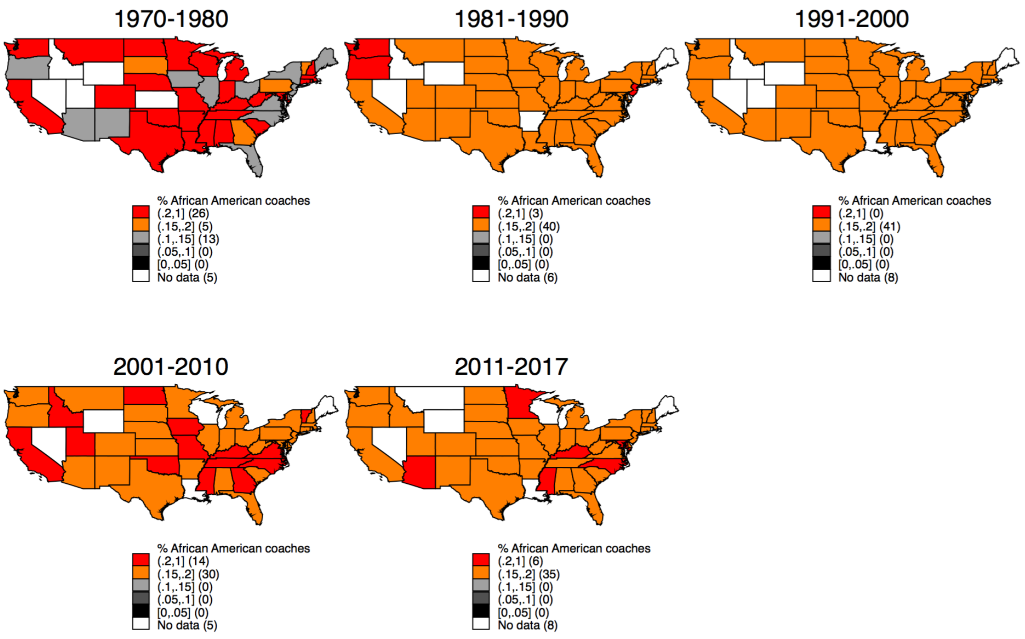

Map 5.2. Coaches in Division 2

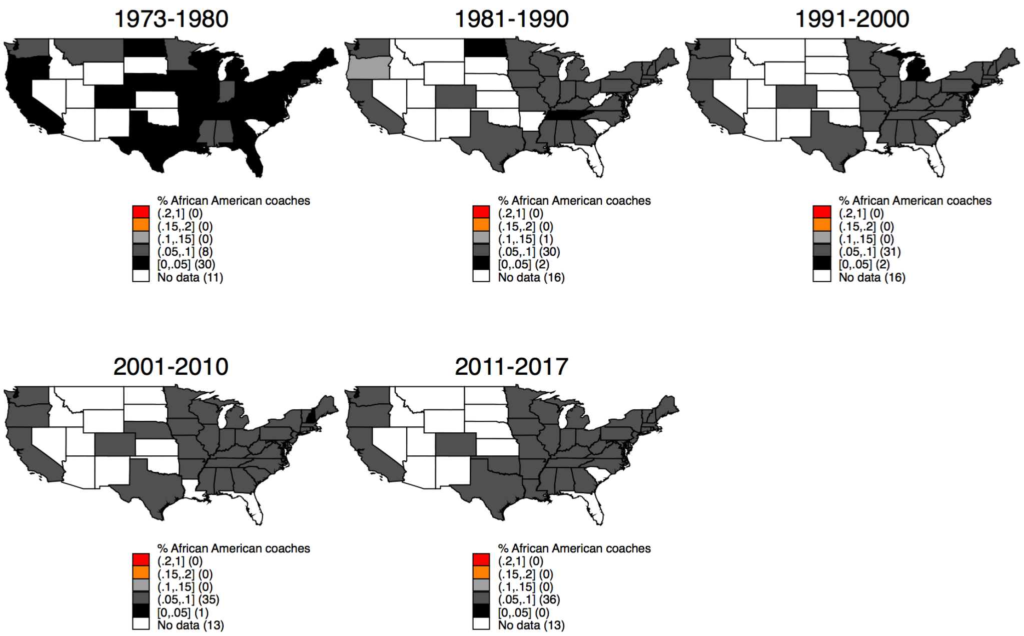

Map 5.3. Coaches in Division 3

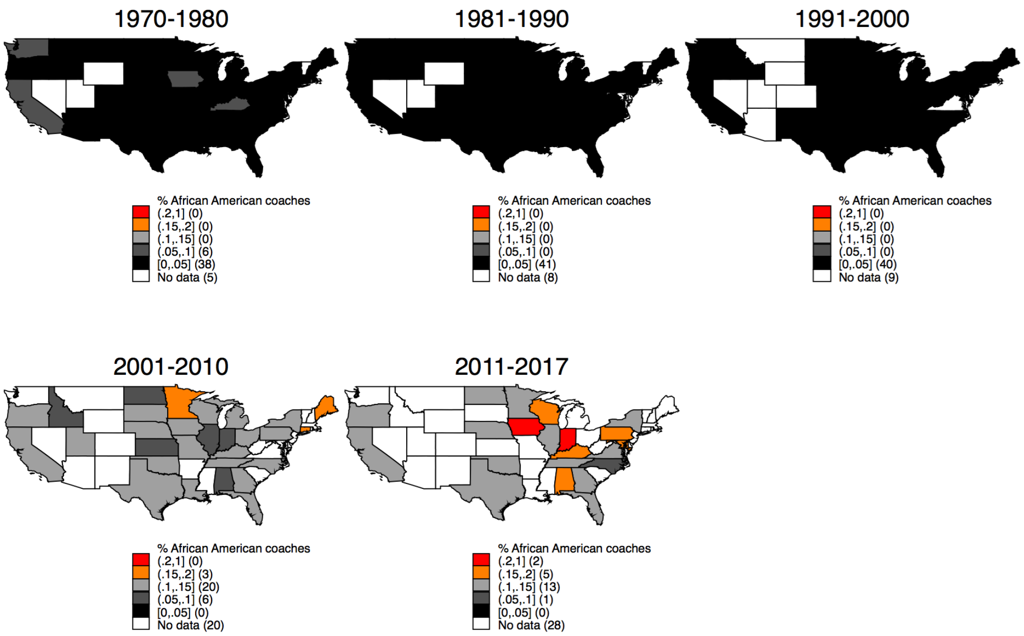

Map 5.4. Coaches outside Division system
